# Dietary lysozyme and avilamycin modulate gut health, immunity, and growth rate in broilers

**DOI:** 10.1186/s12917-023-03871-2

**Published:** 2024-01-20

**Authors:** Mervat A. Abdel-Latif, Hatem S. Abd El-Hamid, Mohamed Emam, Ahmed. E. Noreldin, Yosra A. Helmy, Ali H. El-Far, Ahmed R. Elbestawy

**Affiliations:** 1 Department of Nutrition and Veterinary Clinical Nutrition, Faculty of Veterinary Medicine, Damanhour University, Damanhour, 22511 Egypt; 2 Department of Poultry and Fish Diseases, Faculty of Veterinary Medicine, Damanhour University, Damanhour, 22511 Egypt; 3 Department of Histology and Cytology, Faculty of Veterinary Medicine, Damanhour University, Damanhour, 22511 Egypt; 4 Department of Animal Hygiene, Zoonoses and Animal Ethology, Faculty of Veterinary Medicine, Suez Canal University, Ismailia, 41522 Egypt; 5 Department of Biochemistry, Faculty of Veterinary Medicine, Damanhour University, Damanhour, 22511 Egypt

**Keywords:** Avilamycin, Broilers, Gut health, Lysozyme, Performance

## Abstract

**Background:**

Attempts to use dietary lysozyme (LYZ) as an alternative to antibiotics in broilers have been successful, but further research is needed for effective use. Here, we compared the differences between LYZ and avilamycin (AVI) feed additives for growth performance, gut health and immunity of broilers. One-day old, one hundred and twenty broiler chicks (Ross 308) were randomly allocated into three groups consisting forty birds in each group. Standard diet without supplementation was applied as the control group (I), while the chicks of the other groups were supplemented with 100 mg of AVI per kg diet (AVI, group II), and 90 mg LYZ per kg diet (LYZ, group III) for five consecutive weeks.

**Results:**

Body weight, feed conversion ratio, body weight gain, and European production efficiency factor were markedly (*p* < 0.05) increased in both AVI and LYZ groups in relation to CON group, but the feed intake and protein efficiency ratio were not affected. Both AVI and LYZ significantly (*p* < 0.001) upregulated the mRNA expression of ileal interleukin-18 (*IL-18*), interferon-gamma (*IFN-γ*), and interleukin-10 (*IL-10*), interleukin-2 (*IL-2*), and glutathione peroxidase (*GSH-PX*) genes compared to CON group. However, *IL-2*, *IL-10*, *IL-18*, and *GSH-PX* genes were markedly (*p* < 0.01) upregulated in LYZ compared to the AVI group. LYZ treated group had a significant increase (*p* < 0.05) in the serological haemagglutination inhibition titers of H5N1 vaccination and a significant decrease (*p* < 0.0001) in coliform counts compared to control and AVI groups, but all growth parameters were nearly similar between AVI and LYZ groups. The VH and VH/CD were markedly higher in LYZ than AVI and control groups.

**Conclusion:**

Exogenous dietary lysozyme supplementation by a dose of 90 mg/kg broilers’ diet induced better effects on intestinal integrity, fecal bacterial counts, immune response, and growth performance which were comparable to avilamycin. Therefore, dietary lysozyme could safely replace avilamycin in the broiler chickens’ diet. However, further experimental studies regarding the use of lysozyme in commercial broilers, both in vitro and in vivo, targeting more communities of intestinal microbiome and explaining more details about its beneficial effects need to be conducted.

**Supplementary Information:**

The online version contains supplementary material available at 10.1186/s12917-023-03871-2.

## Introduction

Intestinal integrity in broilers is known as maintaining intestinal health to fully utilize dietary nutrients and allow the expression of full genetic potential for growth. The improvement of this integrity is essential for protection against infectious and non-infectious agents, improve digestion and absorption of nutrients, efficient intestinal microbial balance, and secretion of endogenous materials. All of the previous research strongly suggested that the continuous evaluation of intestinal integrity should be an ongoing activity within a broiler production program. Several antibiotics used in human medicine such as avilamycin, monensin, salinomycin, flavophospholipol have been banned from being added to the animal feed as growth promoters by the USA and/or Europe since January 1^st^ 2006 [1]. The One Health approach necessitates the importance of fighting against antimicrobial resistance (AMR) is one of the top 10 global public health threats facing humanity that was declared by the WHO in 2019. AMR is the silent pandemic or even the silent tsunami, a global threat to public, and animal health and the infections of bacterial origin are the second cause of death worldwide [2].

Thus, searching for antibiotic alternatives for poultry diets with similar One Health benefits is essential [3]. Several probiotics, prebiotics and phytobiotics [4–6], exogenous enzymes [7–9], and organic acids [10] represent promising alternatives to antibiotics. These alternatives have become a mandatory need as a part of the global fight against antibiotic resistance. Lysozyme (LYZ) is one of the most promising antibiotics alternatives. It is an enzyme derived from avian albumin, and is widely distributed in secretions and tissues of animals that plays a vital role against several bacteria that give a positive result in the Gram stain test via hydrolyzing their cell wall’s β-1,4-glycosidic linkage between N-acetyl glucosamine and N-acetyl muramic acid [11, 12]. Following the positive results of our previous study of sole LYZ with different concentrations [13] and others [14], we were motivated to compare the best obtained level of LYZ (90 mg/kg feed) with the most commonly used growth promoter antibiotic AVI (100 mg/kg) [15].

In this way, this novel study was aimed to compare between dietary LYZ and AVI on intestinal health, growth performance and viability in broilers’ production to substitute growth-promoting antibiotics. The outputs of the study can help in combating and limiting the spread of antibiotics resistance in humans to achieve a better way for One Health approach.

## Materials and methods

### Ethical approval

This work was ethically accepted by the Committee of Local Experimental Animal Care, Faculty of Veterinary Medicine, Damanhour University, Egypt (DMU/VetMed-2023/010).

### Experimental design

One-day-old, Ross-308 broiler chicks (obtained from a commercial hatchery), with a total number of 120 were randomly allocated into three groups of mixed sex (40 birds/ group reared in 4 replicates of 10 birds per each). All the 3 chicken groups were caged on wire-floor of equal dimensions [110 (width), 90 (length), 50 (height) cm^3^]. The cage’s mesh was constructed of 7 mm diameter wire and contained 6.25 cm square orifices. Linear feeders were placed outside the cages and sufficient nipple drinkers were supplied. The observation period was 35 days. The incubation temperature was 33 °C and gradually lowered till 25 °C by the 3^rd^ week of age and the duration of light exposure was a 23 h of light daily. The chicks of 3 groups, I (control) fed on a commercial basic diet; II (AVI) supplemented with commercial basic diet containing 100 mg of avilamycin/kg which is the maximum dose recommended by the producer company, Hangzhou Well Sunshine Biotech.Co. Ltd., China, and III (LYZ) had 90 mg of lysozyme 10%/kg diet (Nanchang Lifeng Industry and Trading Co., Ltd., Jiangxi, China) according to Abdel-Latif et al. [12], respectively for 35 days of rearing cycle during April (Spring), 2023. The used corn–soybean based diet were formulated according to the nutrient requirements for Ross-308 broiler chickens [16]. The nutritive analysis of the ingredients was done according to [17]. The calculated composition analysis of the used feed is shown in Table 1. Gradual and effective mixing of both feed supplements was confirmed.

**Table 1:** Ingredients’ percentage and calculated composition analysis of the experimental starter and grower diets (%, as-fed basis)

Ingredients %	Starter (0–10 d)	Grower (11–21 d)	Finisher (22–35 d)
Yellow corn	54.78	58.88	63.90
Soybean meal (44%)	33.5	29.4	24
Corn gluten (60%)	5	5	5
Corn oil	2	2.65	3.15
Dicalcium phosphate	1.73	1.6	1.5
CaCo_3_	1.35	1	1
NaCl	0.4	0.4	0.4
DL-methionine*	0.15	0.12	0.1
HCl-lysine**	0.35	0.3	0.3
Premix (Vitamins and minerals) ***	0.3	0.3	0.3
Toxin binder	0.2	0.2	0.2
Sodium bicarbonate	0.1	0.1	0.1
Choline chloride	0.05	0.05	0.05
Calculated composition			
ME, Kcal/Kg diet	3005	3100	3195
CP%	23	21.5	19.5
Ca%	1	0.87	0.82
Avail. P%	0.47	0.44	0.41
Methionine%	0.56	0.51	0.47
Lysine%	1.44	1.29	1.14
Meth. + Cyst.%	0.93	0.86	0.78
Na%	0.20	0.20	0.20

SBM = soybean meal, ME = metabolizable energy, CP = crude protein, Av. (P) = available phosphorous. * DL—methionine 99% feed grade China. ** L—lysine 99% feed grade. *** Vitamin and mineral premix (Hero mix) produced by Hero pharm and composed (per 3 kg) of vitamin A 12,000,000 IU, vitamin D3 2,500,000 IU, vitamin E 10,000 mg, vitamin K3 2000 mg, vitamin B1 1000 mg, vitamin B2 5000 mg, vitamin B6 1500 mg, vitamin B12 10 mg, niacin 30,000 mg, biotin 50 mg, folic acid 1000 mg, pantothenic acid 10,000 mg, manganese 60,000 mg, zinc 50,000 mg, iron 30,000 mg, copper 4000 mg, iodine 300 mg, selenium 100 mg, and cobalt 100 mg.

### Vaccination

All broilers groups were vaccinated using: (1) inactivated avian influenza subtype H5N1 (AI-H5N1) vaccine (MeFluvac®, MEVAC, Cairo, Egypt) by subcutaneous injection, and bivalent live Newcastle and infectious bronchitis vaccine (Nobilis® Clone 30 + Ma5, MSD, Boxmeer, the Netherlands) on 7 days old, (2) live Gumboro intermediate plus vaccine (Bursine Plus® vaccine, Zoetis Inc. New Jersey, USA) on 14 days old, and (3) live Newcastle vaccine (Nobilis® ND LaSota, MSD, Boxmeer, the Netherlands) on 18 days old. All live vaccines were applied through eye drops.

### Broiler growth performance

Performance parameters including feed intake (FI), body weight (BW), feed conversion ratio (FCR); the ratio between average feed intake and body weight gain, body weight gain (BWG), and protein efficiency ratio (PER) were determined weekly by dividing the number of grams of weight gain produced by weight of dietary protein consumed. Also, mortality rate was recorded throughout the whole experiment. In addition, European production efficiency factor (EPEF) was determined at the end of the experiment using the following equation: [(viability % × body weight Kg / age (d) × FCR)] ×100 [18].

### Haemagglutination inhibition (HI) test

To determine the antibody titers for AI-H5N1, we used standard H5N1 antigen. On the final day, 35, blood samples blood samples (*n* = 5 per group) were collected from the brachial vein of live chickens, centrifuged at 4000 rpm for 15 min (at 4 °C) to get clear sera for the HI test. The positive cut off of samples was the highest serum dilution that caused complete inhibition of 4 haemagglutination units (4 HAU) of the used antigen [19] .

### RNA extraction and RT-PCR

In order to determine the intestinal mRNA genes expression, at the final, 35, day of age, 5 chickens per each group were necropsied after anesthesia through intravenous injection of sodium pentobarbital (50 mg/kg) and the remaining chickens of avilamycin treated group (*n* = 35) were humanly culled using the same method followed by cervical dislocation and hygienic burial. Approximately one cm samples from the ileum (5 cm after the yolk sac diverticulum) were obtained and cleaned with physiological saline (0.75% NaCl) then immersed immediately in the liquid nitrogen (at -80 ^o^C) and stored for further analysis. The total RNA was extracted and purified from intestinal mucosal layer using QIAamp RNeasy Mini kit (Qiagen, GmbH, Germany). The TaqMan RT-PCR was done for *IFN-γ*, *IL-18*, *IL-2*, and *IL10* while QuantiTect SYBR Green PCR Master Mix (Qiagen, GmbH, Germany) was used for the expression of GSHPX (20–24). The primers are listed in supplementary Tables S1 and S2 [20–24]. All values of different genes were normalized to the expression of housekeeping genes, *28 S rRNA* and *β-actin*, using the geometric averaging of multiple internal control genes, fold change = 2^−ΔΔCt^ as previously stated [25].

### Total fecal bacterial count

Fecal samples (*n* = 5), from the same 5 birds in each group, were collected at days 21^st^ and 35^th^, and subjected for total coliform, *Clostridium perfringens* and lactobacillus counts (log_10_ CFU/g). Counts were evaluated based on our previous study [13]. Briefly, ten-fold dilutions (10 ^− 1^ to 10 ^− 7^) of each sample were performed with buffered peptone water and directly inoculated on MacConkey’s agar for total coliform counting and incubated aerobically at 37 °C for 24 h. All red colonies within the range of 15–150 μm were selected for counting.

*C. perfringens* were sub-cultured on Perfringens agar base mixed with 400 mg of D-cycloserine per liter by the dilutions from 10 ^− 1^ to 10 ^− 7^ and incubated anaerobically at 37 °C, using gas generating kits (Oxoid) for 48 h. Plates with black colonies within the range of 25–250 μm were counted. The lactobacillus count was conducted using Rogosa agar plates and cultured by dilutions from 10 ^− 1^ to 10 ^− 7^, then incubated at 37 °C in 5% CO_2_. All whitish colonies that appeared after 48 h of incubation were counted.

### Histology

Jejunal samples (about 3 cm; 1 cm before the midpoint) were collected for histological examination (*n* = 5) from the same 5 birds in each group, washed with saline and rapidly fixed in 10% neutral buffered formalin for 24 h [26]. The fixed samples were paraffin embedded then stained with Hematoxylin and Eosin (H and E) as described by Bancroft and Layton [27]. Sectioning and slide preparation were done according to the protocol described by Saeed et al. [28]. Photos were examined under a light microscope (Leica DM500) at 4× magnification using a digital camera (Leica EC3, Leica, Germany). Measurements of the villi height (VH), villi width in the middle of the individual villus (VW), crypt depth (CD), and VH:CD ratio for each villus in the control and supplemented groups were made by using Image J software (NIH, Bethesda, MD, USA) [29].

### Molecular docking

Molecular docking of AVI against *Gallus gallus* interleukin 1 receptor accessory protein (IL1RAP; AlphaFold ID: A0A1D5P4S3), interleukin 1 receptor type 2 (IL1R2; AlphaFold ID: A0A1L1RVR1), interleukin-1 receptor-associated kinase-like 2 (IRAK2; AlphaFold ID: F1N826), and interleukin-17 receptor D (IL17RD; AlphaFold ID: Q7T2L7) were performed after retrieval of the three dimensional protein structures from AlphaFold database (https://alphafold.ebi.ac.uk/). The protein structures were prepared with BIOVIA Discovery Studio (Vélizy-Villacoublay, France) by removal of water molecules and all ligands in addition to energy minimization and refinement processes. In addition, the 3 dimention structures of AVI were obtained from the PubChem database (https://pubchem.ncbi.nlm.nih.gov/). The scores of molecular docking and the interactions of AVI with prepared proteins were determined using Molecular Operating Environment (MOE) software (Montreal, QC, Canada).

Egg white LYZ (AlphaFold ID: Q6LEL2) were retived from AlphaFold database and prepared with BIOVIA Discovery Studio for molecular docking with *Gallus gallus* IL1RAP, IL1R2, IRAK2, and IL17RD using ClusPro serever (https://cluspro.bu.edu/home.php) for protein-protein docking. Finally, BIOVIA Discovery Studio Visualizer software did the visualization of target-ligands interaction.

### Statistical analysis

All data were statistically analyzed using SPSS (IBM SPSS. 20^®^) utilizing the one-way ANOVA followed by Tukey’s multiple range tests. The data of HI assay, RT-PCR, and total fecal bacterial counts were analyzed by One-way ANOVA, Tukey’s multiple range tests by Graphpad prism 5. The significance was estimated at *p* < 0.05.

## Results

### Broiler growth performance

No marked variations in the chicks’ live BW between different experimental groups were recorded during the first day of age (Table 2). The BW and BWG were markedly (*p* < 0.05) increased by 8, 8.6 and 8.2, 8.8% for AVI and LYZ supplemented groups, respectively, in comparison with control group. Additionally, the FCR and EPEF were markedly (*p* < 0.05) improved in AVI and LYZ groups compared to control group without marked difference between AVI and LYZ groups in all growth measurements. On the other hand, feed intake and PER were unaffected in all chicken groups throughout the whole experimental period, however, they were ameliorated in both supplemented groups in relation to control.

**Table 2:** Effects of avilamycin (AVI) and lysozyme (LYZ) feed supplements on growth performance and economic viability of broilers

Items		Experimental groups			*p*- value
	Control		AVI	LYZ	
Initial weight, g	41.09 ± 0.44		41.11 ± 0.41	41.25 ± 0.39	0.953
^1^fBwt, g	1880.7 ± 40.2^b^		2031.9 ± 39.65^a^	2043.28 ± 39.92^a^	0.012
^2^BWG, g	1839.6 ± 39.9^b^		1990.7 ± 39.4^a^	2002.03 ± 39.63^a^	0.012
^3^FI, g	3084.60 ± 20.4		3142.3 ± 30.7	3155.4 ± 22.96	0.137
^4^FCR	1.69 ± 0.04^a^		1.59 ± 0.03^b^	1.59 ± 0.03^b^	0.035
^5^PER	2.78 ± 0.06		2.95 ± 0.06	2.95 ± 0.06	0.082
^6^EPEF	323.93 ± 14.31^b^		371.98 ± 14.42^a^	374.69 ± 14.47^a^	0.035

Values are given as mean ± Standard error (SE) of four replicates with 10 birds per each.

Any two means for a performance parameter bearing different superscript letters in a row are significantly (*p* < 0.05) different from each other.

^1^Body weight; ^2^Body weight gain; ^3^Feed intake; ^4^Feed conversion ratio; ^5^Protein efficiency ratio and ^6^European production efficiency factor = [(viability % × body weight Kg / age (d) × FCR)] ×100.

### Haemagglutination inhibition (HI) test

**Fig. 1:**
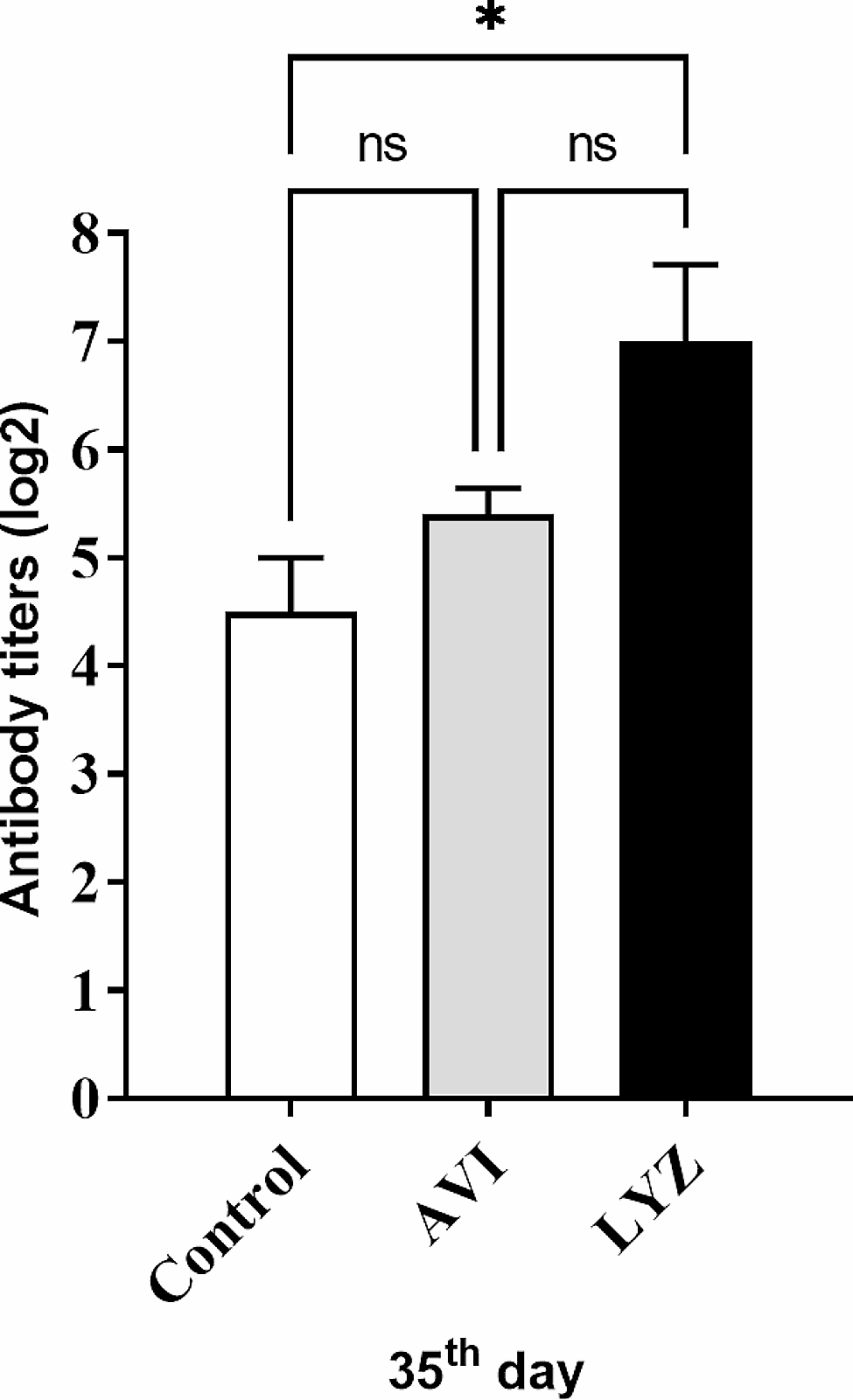
Antibody titer of H5N1 (log _2_) in all groups. Statistical analysis was done by One-way ANOVA, Tukey’s post hoc test multiple comparisons. ns = nonsignificant. *p < 0.05. Error bars represent SE.

Data illustrated in Fig. 1 reveals a significant increase (*p* < 0.05) in serum antibody titers for AI-H5N1 in LYZ-supplemented birds when compared with control and AVI groups, respectively on day 35.

### RNA extraction and RT-PCR

**Fig. 2:**
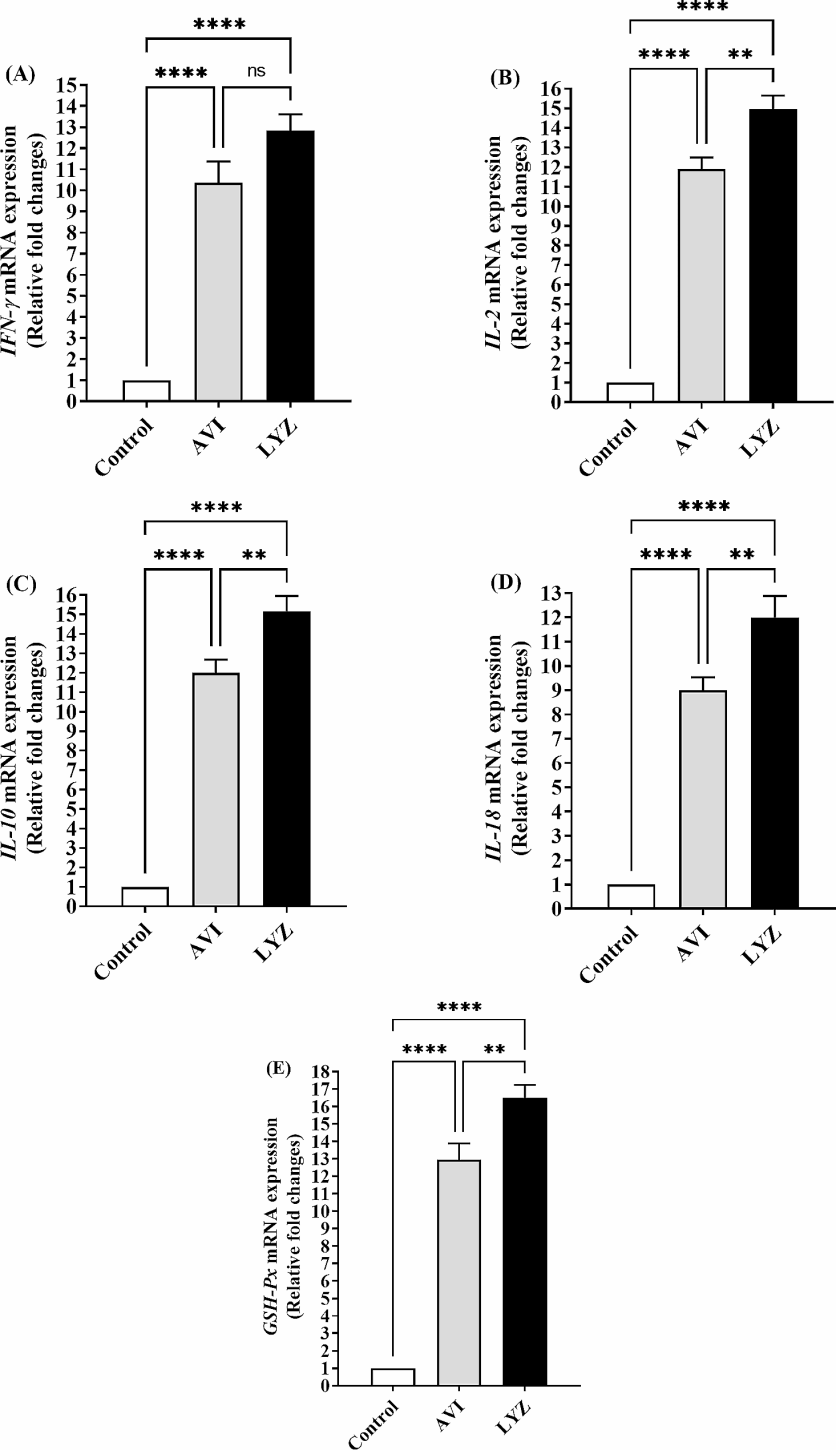
Reverse transcription polymerase chain reaction (RT-PCR) of differentially selected and expressed genes in all groups. Selected genes were *IFN-γ* = interferon-gamma; *IL-2* = interleukin-2, *IL-10* = interleukin-10, *IL-18* = interleukin-18, and *GSH-Px* = glutathione peroxidase. ^*^p < 0.05, ^**^p < 0.01, ^***^p < 0.001, and ^****^p < 0.0001. Statistical analysis was done by One-way ANOVA, Tukey’s *post hoc* test multiple comparisons. Error bars represent SE.

Results illustrated in Fig. 2 which represents the mRNA expression of intestinal interferon-gamma (*IFN-γ*), interleukin-18 (*IL-18*), interleukin-2 (*IL-2*), interleukin-10 (*IL-10*), and glutathione peroxidase (*GSH-PX*) genes. Both AVI and LYZ significantly (*p* < 0.0001) upregulated these genes in comparison with control group. In a comparison between AVI and LYZ groups *IL-2*, *IL-10*, *IL-18*, and *GSH-Px* genes were significantly (*p* < 0.01) upregulated in LYZ compared with AVI group.

### Total fecal bacterial count

**Fig. 3:**
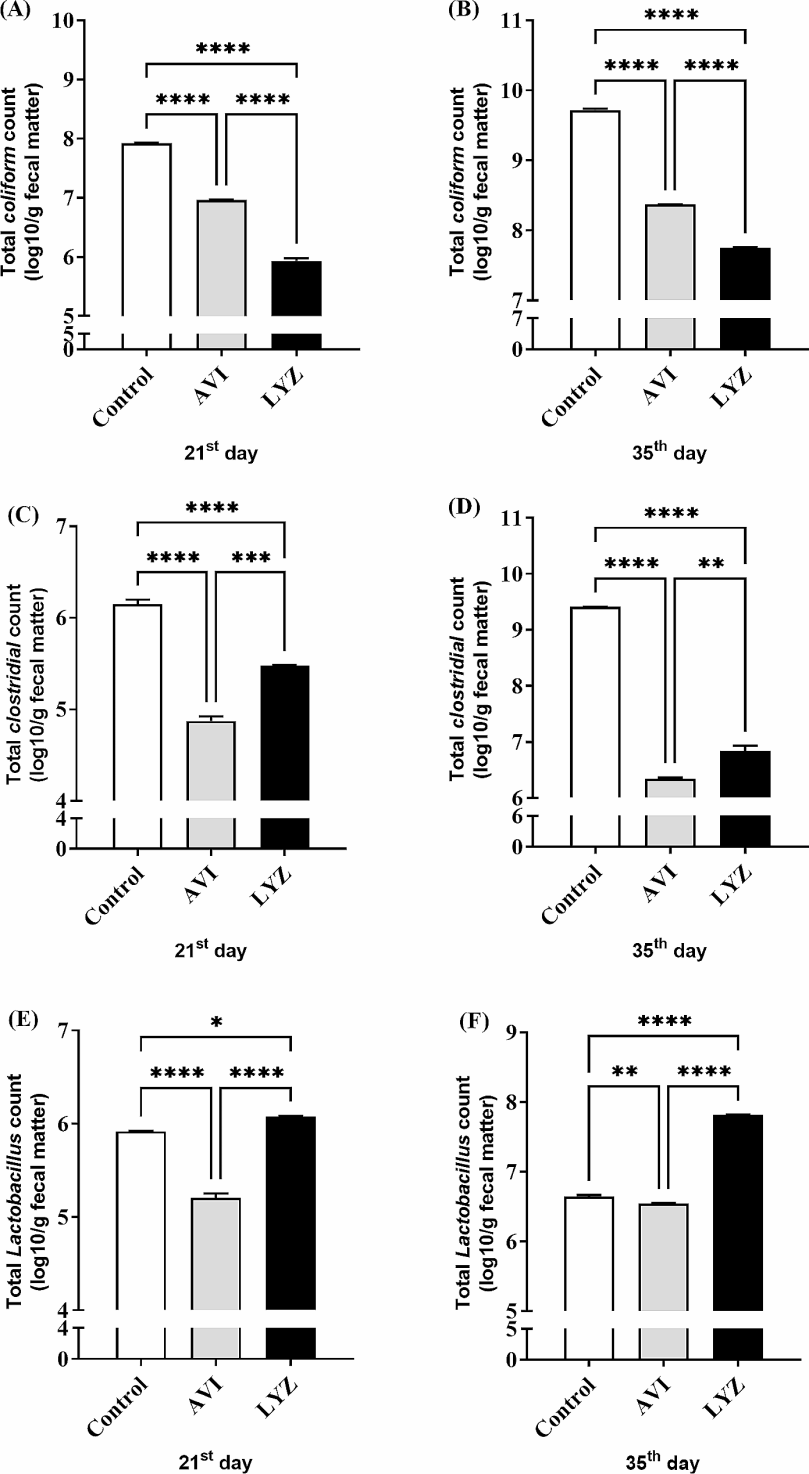
Total coliform **(A, B)**, clostridial **(C, D)**, and lactobacillus **(E, F)** counts in fecal samples. AVI, birds fed avilamycin (100 mg/kg). LYZ, birds fed lysozyme (90 mg/kg). ^*^p < 0.05, ^**^p < 0.01, ^***^p < 0.001, and ^****^p < 0.0001. ns = nonsignificant. Statistical analysis was performed using one-way ANOVA and Tukey’s post hoc test for multiple comparisons

The total coliform count in LYZ supplemented group was lower (*p* < 0.0001) than control or AVI on both 21 and 35 days-old chicks. Regarding total clostridial count, LYZ and AVI supplemented groups had the minimum count (*p* < 0.0001) on 21^st^ and 35^th^ days of age compared to control; however, this count was significantly higher in LYZ in relation to AVI group. Also, LYZ led to the maximum lactobacillus count on 35^th^ days of age (*p* < 0.0001) compared to AVI and control groups, while on 21^st^ days old, LYZ increased the total lactobacillus count with slightly lower significance (*p* < 0.05) (Fig. 3).

### Histology

**Fig. 4:**
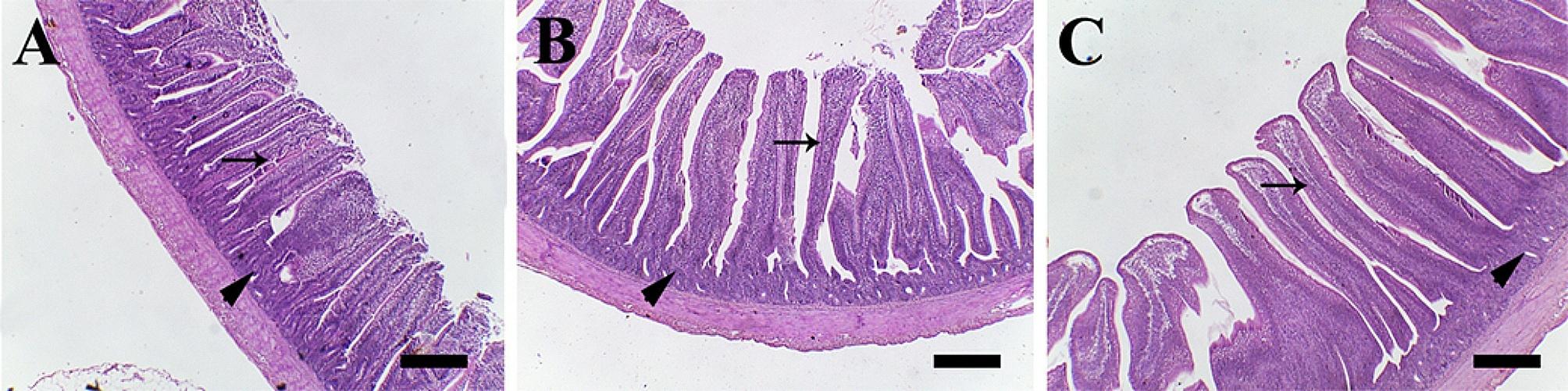
Histological examination of jejunum samples stained with H and E. (**A**) control, (**B**) AVI, and (**C**) LYZ. Arrows point to intestinal villi. Arrowheads point to intestinal crypts. Scale bar = 400 μm

Jejunal histology in response to AVI and LYZ dietary supplements was estimated by measuring villi height (VH), crypt depth (CD), and VH/CD (Tables 3**and** Fig. 4). The height of intestinal villi was markedly higher in LYZ than AVI and control groups. However, the crypt depth of LYZ was similar to that of other groups although it was markedly lower in AVI group than control. As a result, VH/CD was markedly elevated in LYZ and AVI groups respectively, compared with control group.

**Table 3:** Effects of dietary supplementation with avilamycin (AVI) and lysozyme (LYZ) on villi length and crypt depth in broiler chickens

Items	Experimental groups				*p*-value
	Control	AVI	LYZ		
Villus height (VH), µm	1084.97 ± 28.78^b^	1155.19 ± 18.20^b^	1268.39 ± 45.48^a^		0.001
Crypt depth (CD), µm	214.80 ± 8.37^a^	184.34 ± 6.93^b^	198.47 ± 12.49^ab^		0.039
VH/CD	5.20 ± 0.25^b^	6.52 ± 0.24^a^	6.69 ± 0.39^a^		0.001

Values bearing different superscript letters within the same row are significantly (*p* < 0.05) different.

VH, CD and VH/CD analysis were done for 4 replicates (*n* = 10).

### Molecular docking

AVI exhibited binding affinity to IL1RAP, IL1R2, IRAK2, and IL17RD with docking scores of -9.12, -7.32, -8.27, and − 9.19, respectively (Tables 4and Supplementary Fig. 1). AVI interacted with IL1RAP by eight hydrogen (HIS382, VAL383, TRP385, GLU387, ARG393, ARG466, LYS499, and ARG534) and one charge (LEU545) interactions. Similarly, AVI bound by six hydrogen (LYS75, PRO77, SER78, ASP160, ALA162, and LYS250), one charge (LYS75), and three hydrophobic (PHE60, TYR63, and LYS75) with IL1R2. On same context, AVI bound to IRAK2 with more hydrophobic interactions with ARG379, TYR381, MET391, PHE609, and TRP611, while interacted with THR242, ARG379, TYR381, and LYS395 by hydrogen bonds. Also, bound to the binding sites of IRAK2 with four hydrogen (THR242, ARG379, TYR381, and LYS395) and five hydrophobic (ARG379, TYR381, MET391, PHE609, and TRP611) interactions. Finally, AVI also interacted with IL17RD by six hydrogen (GLY43, ARG44, SER47, LYS51, ILE85, and GLU148) and four hydrophobic (ARG44, LYS51, TYR88, and LEU99).

LYZ bound to IL1RAP with ten Pi-interactions and 24 hydrogen bonds as represented in Supplementary File 1 and Supplementary Fig. 2. Similarly, LYZ interacted with IL1R2 by five Pi-interactions and four hydrogen bonds (Supplementary File 2 and Supplementary Fig. 2). Interaction between LYZ and IRAK2 exhibited one salt bridge, eight Pi-interactions, and 15 hydrogen bonds (Supplementary File 3 and Supplementary Fig. 2). Also, by one salt bridge, 14 Pi-interactions, and 31 hydrogen bonds (Supplementary File 4 and Supplementary Fig. 2), LYZ was bound to IL17RD.

**Table 4:** Molecular scores and interactions of avilamycin (AVI) against *Gallus gallus* interleukin 1 receptor accessory protein (IL1RAP; AlphaFold ID: A0A1D5P4S3), interleukin 1 receptor type 2 (IL1R2; AlphaFold ID: A0A1L1RVR1), interleukin-1 receptor-associated kinase-like 2 (IRAK2; AlphaFold ID: F1N826), and interleukin-17 receptor D (IL17RD; AlphaFold ID: Q7T2L7)

	Docking scores	Molecular interactions
IL1RAP	-9.12	Hydrogen: HIS382, VAL383, TRP385, GLU387, ARG393, ARG466, LYS499, and ARG534. Charge: LEU545
IL1R2	-7.32	Hydrogen: LYS75, PRO77, SER78, ASP160, ALA162, and LYS250 Charge: LYS75 Hydrophobic: PHE60, TYR63, and LYS75
IRAK2	-8.27	Hydrogen: THR242, ARG379, TYR381, and LYS395 Hydrophobic: ARG379, TYR381, MET391, PHE609, and TRP611
IL17RD	-9.19	Hydrogen: GLY43, ARG44, SER47, LYS51, ILE85, and GLU148 Hydrophobic: ARG44, LYS51, TYR88, and LEU99

## Discussion

It is well known that broilers are the highest efficient land animals in converting energy into meat [30]. However, the broiler performance (BW, BWG, FCR, and PER) is closely related to the intestinal integrity and the microbiome community of bird gut. Several investigations in chickens have revealed the significance of intestinal integrity and gut microbiota on some pivotal functions such as mucosal immunity, gut development, nutrient absorption and digestion of feed by the host [31–35]. In the same context, LYZ supplement to broiler chickens diet (90 mg/kg) amended the bird performance and gut health [13, 14]. In the current study, both LYZ and AVI supplementation significantly improved performance parameters including BW, BWG, FCR, and EPEF, with the absence of significant difference in FI and PER compared with control group. Furthermore, the molecular docking model showed high affinity of either AVI or LYZ to bind with IL1RAP, IL1R2, IRAK2, and IL17RD, which indicated that AVI and LYZ could bind to the intestinal IL1RAP, IL1R2, IRAK2, and IL17RD leading to subsequent diminishing of intestinal inflammatory process. These anti-inflammatory effects of AVI or LYZ led to improve the gut health which might reflect on the general performance of the bird. These computational results need future biological assessment to investigate the effects of AVI and LYZ on these target proteins synthesis and interactions.

In this study, the performance enhancement may be attributed to the improved gut morphology that was explained by significant higher VH and VH/CD ratio in jejunum of LYZ group compared to other groups despite similar crypt depth in control vs. LYZ group and in LYZ vs. AVI. In addition, higher value of LYZ group compared to AVI group, however, crypt depth was significantly high in control group compared to AVI group (Table 3; Fig. 4). The increase in VH is was previously suggested to be a gut absorption efficiency indicator [36, 37]. The improved VH and VH/CD ratio lead to enhancement of nutrient absorption rates in the small intestine [38, 39]. It is well known that diet has the major impact on the intestinal morphology and its absorptive capacity which reflects on the broiler growth performance [40]. So, longer intestinal villi means more intestinal absorptive area and more intestinal absorptive capacity for nutrients and more growth [41].

Intestinal crypts are the main source of the intestinal stem cells to renew and regenerate intestinal villi. So, deeper intestinal crypts leads to more production of intestinal villi cells and more absorption [40] and decreasing crypt depths results in reduced nutrient absorption [42]. Consequently, our results in this research confirmed the explanation of the role of LYZ in changing gut morphology and modulation of some gut microbiota in our previous study [12]. Also, LYZ supplementation significantly decreased the total coliform count and significantly increased the total lactobacillus count at 21^st^ and 35^th^ days of age compared with other 2 groups which might lead to improved eubiosis. The total clostridial count in LYZ group was significantly lower than control group, however, it was higher (*p* < 0.01 − 0.001) than AVI supplemented group. Some investigations had recorded marked effect of LYZ in different organisms as a good defense facing pathogenic bacteria of both Gram types [43, 44]. The upregulation in *IFN-γ*, which is a mediator of mucus that has antimicrobial roles, a physical barrier, as well as being composed of mucin glycoproteins that enhance the growth of beneficial bacteria and have direct toxic to many harmful bacteria might have a role in the significant increase of lactobacillus, and significant decrease in coliform, and clostridial counts [13, 45].

Modulation of some intestinal microbiota such as total coliform, clostridial, and lactobacillus counts resulted in the enhancement of intestinal immune system and its pro- and anti-inflammatory status where the expression of intestinal *IFN-γ*, *IL-18*, *IL-2*, *IL-10*, and *GSH-Px* genes in the present study were more upregulated in LYZ supplemented group. The intestinal tract is the main entrance for several bacteria and viruses. Mucosal adaptive and innate immune cells are prepared to fight invading pathogens [37, 46]. IL-10, produced by epithelial cells is critical for lowering the gut inflammation. Also, the enhanced IL-2, stimulate the proliferation and cytotoxicity via intestinal lymphocytes [47]. IL-2, maintaining the integrity of in rat intestinal epithelial cell lines [48]. IL-18 a pro-inflammatory mediator in several cell types which has important roles in enhancing the production of IFN-γ, induction the differentiation of Th1 cells and priming of NK cell cytotoxicity [49, 50]. The enhanced IFN-γ protects gut against bacterial infections [46]. *GSH-Px* gene encodes GSH-Px (EC 1.11.1.9) enzyme that is a member of endogenous antioxidant enzymatic defense against different xenobiotics. After H_2_O_2_ has been produced by the action of superoxide dismutase (SOD), it is reduced to water by GSH-Px and catalase [51]. GSH-Px is located in the epithelial cells of GIT where it have a role in reduction of dietary peroxides [52].

Moreover, it is worth mentioning that LYZ supplementation has a pivotal role in the stimulation of cellular and humoral immunity [53]. Also, Jiménez-Saiz et al. [54] and Mine et al. [55] stated that oral lysozyme could be hydrolyzed in the duodenum and be accompanied by the production of antimicrobial peptides that could play critical roles in innate immunity. Herein, a significant increase in serum antibody titers for AI-H5N1 in LYZ-supplemented G3 than in control and AVI groups, respectively at 35 days suggested the immunomodulatory effect of LYZ. Furthermore, van de Crommenacker et al. [56] detected that LYZ supplementation in adult pigeons augmented immune function.

## Conclusions

Exogenous dietary lysozyme supplementation to broilers with a dose of 90 mg/kg had better comparable effects on intestinal health, fecal bacterial counts, growth performance and immune response to avilamycin. Therefore, it is recommended to use this promising alternative, lysozyme, to avilamycin achieving the goals of one health approach. However, further in vitro and in vivo experimental studies regarding the use of lysozyme in broilers are needed to target more communities of intestinal microbiome and explore more details about its beneficial effects.

### Electronic supplementary material

Below is the link to the electronic supplementary material.

Supplementary Material 1

Supplementary Material 2

Supplementary Material 3

Supplementary Material 4

Supplementary Material 5. **Supplementary Fig. 1.** Molecular scores and interactions of avilamycin (AVI) against Gallus gallus in-terleukin 1 receptor accessory protein (IL1RAP; AlphaFold ID: A0A1D5P4S3), interleukin 1 receptor type 2 (IL1R2; AlphaFold ID: A0A1L1RVR1), interleukin-1 receptor-associated kinase-like 2 (IRAK2; AlphaFold ID: F1N826), and interleukin-17 receptor D (IL17RD; AlphaFold ID: Q7T2L7)

Supplementary Material 6. **Supplementary Fig. 2.** Molecular scores and interactions of lysozyme (LYZ) against Gallus gallus in-terleukin 1 receptor accessory protein (IL1RAP; AlphaFold ID: A0A1D5P4S3), interleukin 1 receptor type 2 (IL1R2; AlphaFold ID: A0A1L1RVR1), interleukin-1 receptor-associated kinase-like 2 (IRAK2; AlphaFold ID: F1N826), and interleukin-17 receptor D (IL17RD; AlphaFold ID: Q7T2L7)

Supplementary Material 7. **Table S1** Primers sequences, target genes and cycling conditions for TaqMan RT-PCR. **Table S2** Primers sequences, target genes and cycling conditions for SYBR green RT-PCR.

## Data Availability

The data presented in this study are available on request from the corresponding authors.

## Abbreviations

ANOVA: Analysis of variance
Avail. P: Available phosphorus
BW: Body weight
BWG: Body weight gain
CD: Crypt depth
CP: Crude protein
EPEF: European production efficiency factor
FCR: Feed conversion ratio
GSH-Px: Glutathione peroxidase
IFN-γ: Interferon-gamma
IL: Interleukin
ME: Metabolizable energy
MOE: Molecular Operating Environment
PER: Protein efficiency ratio
RT-PCR: Reverse transcription polymerase chain reaction
FI: Feed intake
VH: Villi height
