# Supplementary material for: Dietary lysozyme and avilamycin modulate gut health, immunity, and growth rate in broilers
Source: BMC Vet Res. 2024 Jan 20;20:28. doi: 10.1186/s12917-023-03871-2 (PMC10799510; doi:10.1186/s12917-023-03871-2)
Supplement: Supplementary file 6 — Supplementary Material 6. Supplementary Fig. 2. Molecular scores and interactions of lysozyme (LYZ) against Gallus gallus in-terleukin 1 receptor accessory protein (IL1RAP; AlphaFold ID: A0A1D5P4S3), interleukin 1 receptor type 2 (IL1R2; AlphaFold ID: A0A1L1RVR1), interleukin-1 receptor-associated kinase-like 2 (IRAK2; AlphaFold ID: F1N826), and interleukin-17 receptor D (IL17RD; AlphaFold ID: Q7T2L7) [file 12917_2023_3871_MOESM6_ESM.pptx]

## Slide 1
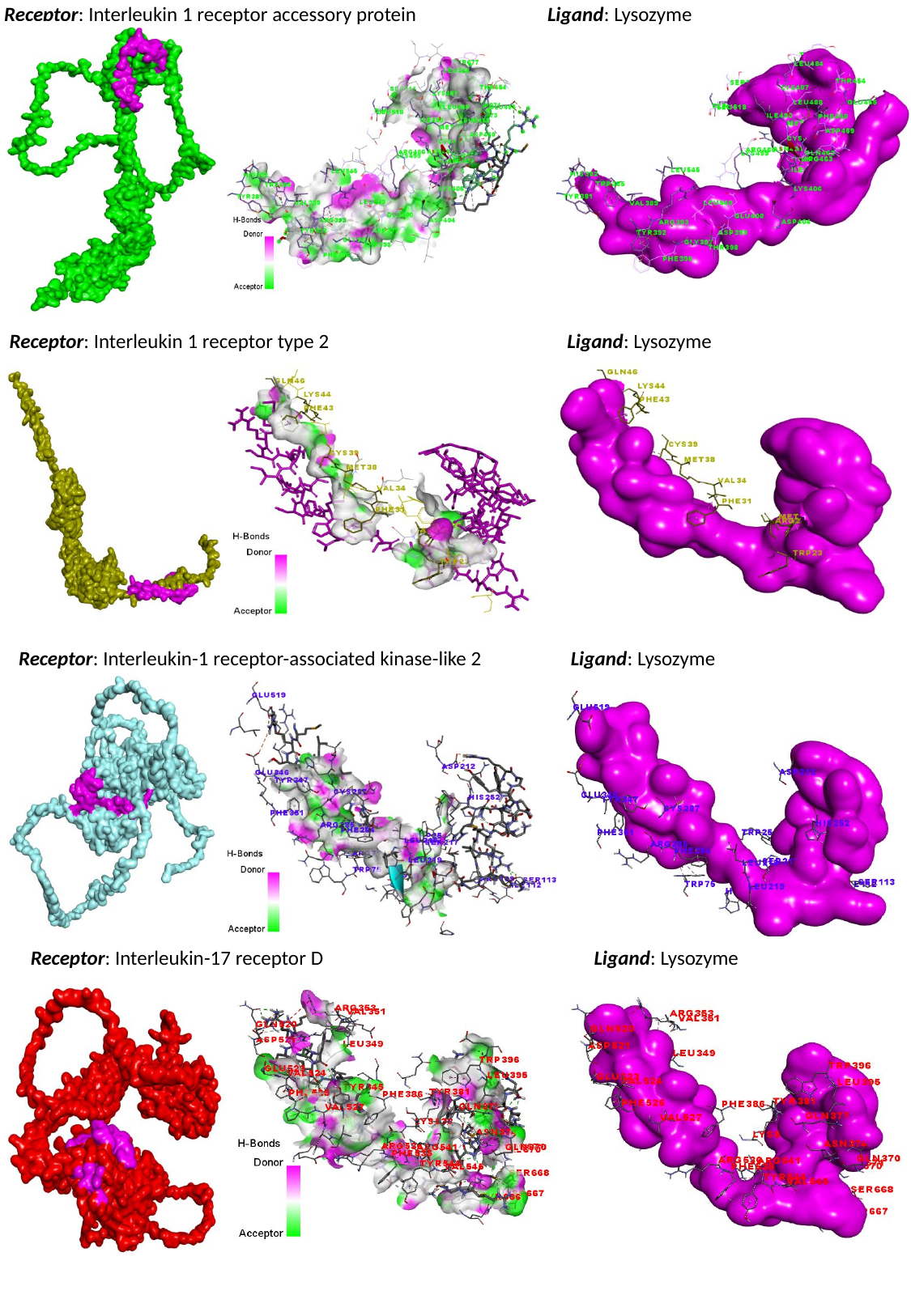

Receptor: Interleukin 1 receptor accessory protein Ligand: Lysozyme
Receptor: Interleukin 1 receptor type 2 Ligand: Lysozyme
Receptor: Interleukin-1 receptor-associated kinase-like 2 Ligand: Lysozyme
Receptor: Interleukin-17 receptor D Ligand: Lysozyme
