# Supplementary material for: Dietary lysozyme and avilamycin modulate gut health, immunity, and growth rate in broilers
Source: BMC Vet Res. 2024 Jan 20;20:28. doi: 10.1186/s12917-023-03871-2 (PMC10799510; doi:10.1186/s12917-023-03871-2)
Supplement: Supplementary file 7 — Supplementary Material 7. Table S1 Primers sequences, target genes and cycling conditions for TaqMan RT-PCR. Table S2 Primers sequences, target genes and cycling conditions for SYBR green RT-PCR. [file 12917_2023_3871_MOESM7_ESM.docx]

**Table S1** Primers sequences, target genes and cycling conditions for TaqMan RT-PCR

| **Target gene** | **Primers and probes sequences**  **(5'-3')** | **Reverse**  **transcription** | **Primary**  **denaturation** | **Amplification (40 cycles)** | | **References** |
| --- | --- | --- | --- | --- | --- | --- |
|  |  |  |  | **Secondary denaturation** | **Annealing and extension**  **(Optics on)** |  |
| *28S rRNA* | F: GGCGAAGCCAGAGGAAACT | 50˚C  30 min. | 94˚C  5 min. | 94˚C  15 sec. | 60˚C  1 min. | [20] |
|  | R: GACGACCGATTTGCACGTC |  |  |  |  |  |
|  | (FAM) AGGACCGCTACGGACCTCCACCA (TAMRA) |  |  |  |  |  |
| *IFN-γ* | F: GTGAAGAAGGTGAAAGATATCATGGA |  |  |  |  |  |
|  | R: GCTTTGCGCTGGATTCTCA |  |  |  |  |  |
|  | (FAM) GGCCAAGCTCCCGATGAACGA (TAMRA) |  |  |  |  |  |
| *IL-18* | F: AGGTGAAATCTGGCAGTGGAAT |  |  |  |  |  |
|  | R: ACCTGGACGCTGAATGCAA |  |  |  |  |  |
|  | (FAM) CCGCGCCTTCAGCAGGGATG (TAMRA) |  |  |  |  |  |
| *IL-2* | F: TTGGAAAATATCAAGAACAAGATTCATC |  |  |  | 59˚C  1 min. | [21] |
|  | R: TCCCAGGTAACACTGCAGAGTTT |  |  |  |  |  |
|  | (FAM) ACTGAGACCCAGGAGTGCACCCAGC (TAMRA) |  |  |  |  |  |
| *IL-10* | F: CATGCTGCTGGGCCTGAA |  |  |  | 60˚C  1 min. | [22] |
|  | R: CGTCTCCTTGATCTGCTTGATG |  |  |  |  |  |
|  | (FAM) CGACGATGCGGCGCTGTCA (TAMRA) |  |  |  |  |  |

**Table S2** Primers sequences, target genes and cycling conditions for SYBR green RT-PCR

| **Target gene** | **Primers sequences**  **(5'-3')** | **Reverse transcription** | **Primary**  **denaturation** | **Amplification (40 cycles)** | | | **References** |
| --- | --- | --- | --- | --- | --- | --- | --- |
|  |  |  |  | **Secondary denaturation** | **Annealing**  **(Optics on)** | **Extension** |  |
| *ß. Actin* | F: CCACCGCAAATGCTTCTAAAC | 50˚C  30 min. | 94˚C  5 min. | 94˚C  15 sec. | 60˚C  30 sec. | 72˚C  30 sec. | [[23](#_ENREF_22)] |
|  | R: AAGACTGCTGCTGACACCTTC |  |  |  |  |  |  |
| *GSH-PX* | F: TTGTAAACATCAGGGGCAAA |  |  |  | 60˚C  30 sec. |  | [24] |
|  | R: ATGGGCCAAGATCTTTCTGTAA |  |  |  |  |  |  |
